# Cytokine-primed umbilical cord mesenchymal stem cells enhanced therapeutic effects of extracellular vesicles on osteoarthritic chondrocytes

**DOI:** 10.3389/fimmu.2022.1041592

**Published:** 2022-10-27

**Authors:** Thu Huyen Nguyen, Huy Hoang Dao, Chau Minh Duong, Xuan-Hung Nguyen, Diem Huong Hoang, Xuan-Hai Do, Trung Quang Truong, Tu Dac Nguyen, Liem Thanh Nguyen, Uyen Thi Trang Than

**Affiliations:** ^1^ Center of Applied Sciences, Regenerative Medicine and Advance Technologies, Vinmec Healthcare System, Hanoi, Vietnam; ^2^ Faculty of Biology, VNU University of Science, Vietnam National University, Hanoi, Vietnam; ^3^ Department of Biology, Clark University, Worcester, MA, United States; ^4^ College of Health Sciences, VinUniversity, Hanoi, Vietnam; ^5^ Department of Practical and Experimental Surgery, Vietnam Military Medical University, Hanoi, Vietnam; ^6^ Hanoi Medical University, Hanoi Medical University Hospital, Hanoi, Vietnam; ^7^ Vinmec Research Institute of Stem Cell and Gene Technology, Vinmec Healthcare System, Hanoi, Vietnam

**Keywords:** osteoarthritis, chondrocytes, mesenchymal stem cells, extracellular vesicles, cytokines, microRNA

## Abstract

In recent years, extracellular vesicles (EVs) secreted by mesenchymal stem cells (MSCs) have emerged as a potential cell-free therapy against osteoarthritis (OA). Thus, we investigated the therapeutic effects of EVs released by cytokine-primed umbilical cord-derived MSCs (UCMSCs) on osteoarthritic chondrocyte physiology. Priming UCMSCs individually with transforming growth factor beta (TGFβ), interferon alpha (IFNα), or tumor necrosis factor alpha (TNFα) significantly reduced the sorting of miR-181b-3p but not miR-320a-3p; two negative regulators of chondrocyte regeneration, into EVs. However, the EV treatment did not show any significant effect on chondrocyte proliferation. Meanwhile, EVs from both non-priming and cytokine-primed UCMSCs induced migration at later time points of measurement. Moreover, TGFβ-primed UCMSCs secreted EVs that could upregulate the expression of chondrogenesis markers (*COL2* and *ACAN*) and downregulate fibrotic markers (*COL1* and *RUNX2*) in chondrocytes. Hence, priming UCMSCs with cytokines can deliver selective therapeutic effects of EV treatment in OA and chondrocyte-related disorders.

## Introduction

Extracellular vesicles (EVs) are nano-sized, lipid membrane-enclosed particles that modulate the physiological conditions of the recipient cells (1). By effectively delivering a wide range of bioactive molecules involved in critical signaling pathways associated with apoptosis, proliferation, migration, extracellular matrix (ECM) synthesis, cartilage regeneration, and inflammation management, EVs have been studied for their therapeutic effects on several cartilage-related diseases (2). Recently, multiple approaches have been employed to enhance therapeutic effect, and targetable EV delivery, including engineering secreted cells, loading therapeutic molecules into naturally secreted vesicles (3), and conjugating the vesicles with targeting ligands (4). Additionally, the therapeutic cargo of EVs secreted by mesenchymal stem cells (MSCs) varies depending on MSC tissue sources (5). Since MSCs are very sensitive to environmental conditions, priming these cells with cytokines as supplements in the culture media can influence bioactive molecules packed in the derived EVs, thereby affecting the biological activities of the vesicles (6, 7).

In this study, we primed MSCs originated from the umbilical cord (UCMSCs) with anti-inflammatory cytokines (transforming growth factor beta - TGFβ and interferon alpha - IFNα) and inflammatory cytokines (including tumor necrosis factor alpha - TNFα), which are linked with osteoarthritis (OA) pathogenesis. In healthy cartilage, TGFβ stimulates chondrocyte proliferation while suppressing chondrocyte hypertrophy and maturation, as well as promoting chondrocytes to synthesize ECM components (8). Additionally, the inhibition of TGFβ signaling leads to chondrocyte terminal differentiation and the early onset of OA (9). Another stimulant in the anti-inflammatory, IFNα, plays a vital role in autoimmunity and inflammation and could effectively protect against antigen-induced arthritis by inhibiting pro-inflammatory cytokine (interleukin 1β (IL-1β), IL-6, IL-17, TNF, IL-12, and IFNγ) production while inducing TGFβ synthesis (10). Moreover, injection of IFNα into the synovial fluid promotes the generation of functional antagonists such as interleukin 1 receptor antagonist (IL-1Ra), soluble tumor necrosis factor receptors (sTNFR), and osteoprotegerin (OPG) for known OA-inducing factors of IL-1, TNF, and osteoprotegerin ligand (11). However, direct administration of these two cytokines to patients frequently harms the nearby tissues, such as a synovial membrane or subchondral bone, as well as general health, including headache, malaise, fever, and even depression (12). The inflammatory cytokine TNFα which is one crucial catabolic factor for cartilage, promotes synovial fibroblasts to release Matrix metalloproteinase (MMPs), resulting in cartilage destruction during OA progression (13). This cytokine is also able to signal chondrocyte apoptosis leading to a more severe OA phenotype (13). Moreover, priming UCMCS with cytokines enhances the anti-inflammatory and immunomodulatory potential of the secreted EVs (14–16). TNFα stimulation was shown to induce the expression of immunosuppressive factors in the parental MSCs, which produced exosomes that can modulate M2/M1 macrophage differentiation (14). The molecular content changes in EV derived from cytokine-stimulated MSCs can interfere with inflammation *via* PGE2/COX10 mechanism (15). Although the immunomodulatory effect of EVs from cytokines-primed cells in the context of OA has been reported before (14, 17), no publication was found indicating their influence on chondrocytes.

In OA, several miRNAs found in EVs have been demonstrated to regulate key signaling pathways involved in ECM maintenance, chondrocyte proliferation, migration, apoptosis, and inflammation (2). Thus, modulating miRNA composition might directly influence the EV therapeutic effect. In this study, we focused on two candidate miRNAs, miR-320a-3p and miR-181b-3p, which involve in cartilage homeostasis. Previous studies showed that miR-320a played essential roles in the secretion of matrix degradation factors (18) and chondrocyte proliferation (19) in OA models. Although not many studies focused on miR-181b-3p, it was described to inhibit proliferation as well as promote apoptosis of chondrocytes in OA (20).

As described, different EV sub-populations carry a different set of bioactive molecules (21), for instance, miRNAs content; thus, we assumed that they would have a distinct impact on chondrocytes. We further hypothesized that priming UCMSCs with the cytokines could alter the miR-181b-3p and miR-320a-3p levels in the secreted EVs, thereby modulating the effect of EVs in chondrocytes proliferation, migration, and their markers.

## Results

### Cytokine-primed UCMSCs and their EVs expressed regular morphology and molecular markers

**Figure 1:**
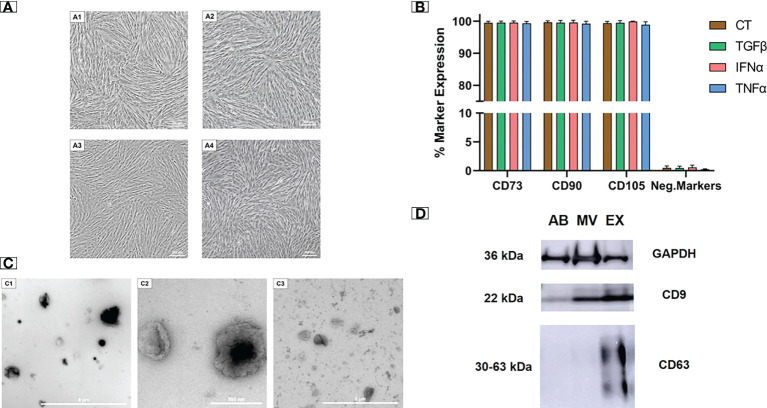
Cytokine-primed UCMSCs and their EVs expressed typical morphology and molecular markers. **(A1 - A4)** Typical morphology of **(A1)** non-priming UCMSCs; **(A2)** TGFβ-primed UCMSCs; **(A3)** IFNα-primed UCMSCs; **(A4)** TNFα-primed UCMSCs; were captured under Nikon Inverted Microscope Eclipse Ti-S at day 5 of P5. **(B)** Expression of MSC markers (n = 3) was analyzed using the flow cytometry approach. **(C1 - C3)** Morphology of different EV populations was observed under TEM. **(C1)** An AB representative with a diameter ranging from 500 nm to 2000 nm (scale bar 9 μm); **(C2)** A MV representative with irregular shapes and diameters from 200 nm to 400 nm (scale bar 500 nm); **(C3)** An EX representative that has cup-shaped morphology and sizes ranging from 40 to 200 nm (scale bar 2 µm). **(D)** A representative of EV markers (22). 15 μg total EV protein was loaded in each lane. Internal reference GAPDH and CD9 were detected in all three EV populations. CT, non-priming UCMSCs; TGFβ, TGFβ-primed UCMSCs; IFNα, IFNα-primed UCMSCs; TNFα, TNFα-primed UCMSCs. Apoptotic Bodies; MV, Microvesicles; EX, Exosomes. All representative images of EVs were obtained from EVs secreted by TGFβ-primed UCMSCs. Error bars indicate ± SD.

We examined the morphology and cell surface markers of UCMSCs at passage 5 (P5), either non-priming or cytokine priming. We observed a typical UCMSC morphology with a spindle shape in both control (EV-depleted) and cytokine-primed (TGFβ, IFNα, TNFα) culture conditions (**Figures 1A1-A4**). Additionally, all cultured UCMSCs expressed MSC positive markers of CD90, CD105, and CD73 (> 95%). Meanwhile, MSC negative markers of CD45, CD34, CD11b, CD19, and HLA-DR were detected with low percentages (< 2%) (**Figure 1B**). Hence, cytokine priming did not alter the typical morphology and surface markers of UCMSCs.

EVs isolated from UCMSC culture medium were subjected to morphology analysis by transmission electron microscope (TEM). Three EV sub-populations including apoptotic bodies (ABs), microvesicles (MVs), and exosomes (EXs), were observed with distinguished shapes and sizes (**Figures 1C1 - C3**). ABs showed variable shapes with a diameter scale of approximately 500 nm to 2000 nm, and they are packed within a rough membrane (**Figure 1C1**). MVs had variable membrane-bound morphologies with uneven surfaces and diameters ranging from 100 nm to 1 µm (**Figure 1C2**). EXs exhibited a typical cup-shaped morphology with their size ranging from 40 to 200 nm (**Figure 1C3**).

Additionally, the isolated EVs expressed standard EV protein markers (CD9 and CD63). As a control indicator, all three EV populations strongly expressed the internal reference protein of GAPDH. For general EV marker expression, CD9 was present abundantly in MVs and EXs and lightly in ABs. EXs also expressed a typical exosomal marker of CD63, which was absent in MVs and ABs (**Figure 1D**). The morphology and protein marker analysis confirmed the identity of three separated EV populations from UCMSC conditioned media.

### Differential levels of microRNA 320a-3p and 181b-3p into EVs secreted by cytokine-primed UCMSCs

**Figure 2:**
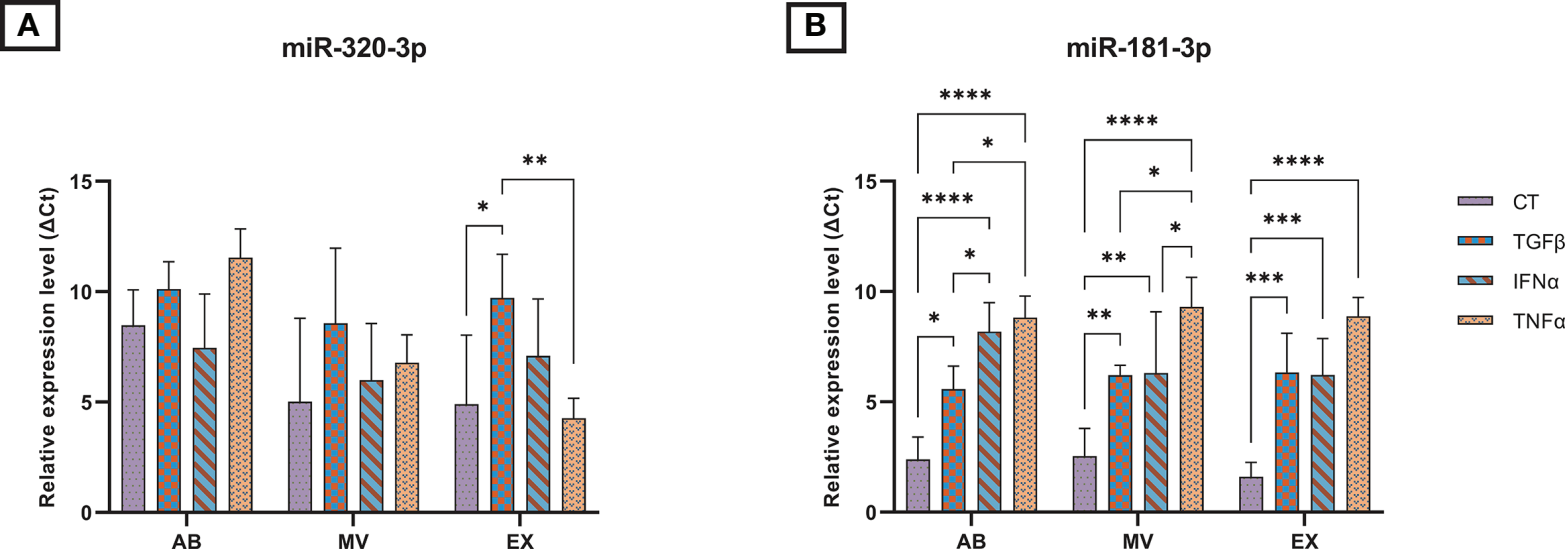
The relative expression of microRNAs in three EV populations in control and inducing groups. The miRNA expression levels in EVs are represented by ΔCt values with 7.5 ng cDNA input and were normalized to the secreted cells (UCMSCs). The relative expression level in different EV sub-populations of **(A)** miR-320a-3p; **(B)** miR-181b-3p. Cytokines priming suppressed the selective sorting of miR-181b-3p but not miR-320a-3p into UCMSC-derived EVs. AB: Apoptotic bodies; MV: Microvesicles; EX: Exosomes. CT-AB/MV/EX: AB/MV/EX secreted from non-priming UCMSCs; TGFβ-AB/MV/EX: AB/MV/EX secreted from TGFβ-primed UCMSCs; IFNα-AB/MV/EX: AB/MV/EX secreted from IFNα-primed UCMSCs; TNFα-AB/MV/EX: AB/MV/EX secreted from TNFα-primed UCMSCs. Results were averages of 3 biological samples (n = 3). Statistical significance was determined by Two-Way ANOVA and indicated by: * where *p* < 0.05; ** where *p* < 0.01; *** where *p* < 0.001; **** where *p* < 0.0001. Error bars indicate ± SD.

We measured the expression of selected miRNAs associated with OA pathogenesis present in UCMSCs and three secreted EV populations (ABs, MVs, and EXs) from normal and cytokine-primed conditions. Using qRT-PCR, we quantified the levels of two candidate miRNAs: miR-181b-3p and miR-320a-3p. Generally, both miR-181b-3p and miR-320a-3p were detected in all UCMSCs and isolated EV sub-populations of ABs, MVs, and EXs from different culture conditions (**Figure 2** and **Supplementary Figure 1**).

In UCMSCs, cytokine treatments acted differentially on the expression of two candidate miRNAs. Particularly, TGFβ and TNFα significantly induced the levels of miR-320a-3p present in UCMSCs, indicated by lower delta Ct values (**Supplementary Figure 1A**). Besides, no significant impact was detected on the expression of miR-181b-3p (**Supplementary Figure 1B**).

In contrast, cytokine-priming significantly modulated the levels of miR-181b-3p while producing a little impact on the levels of miR-320-3p packed into EVs. Cytokine treatment suppressed the selective sorting of miR-181b-3p in all three EV sub-populations compared to the non-priming group, indicated by higher delta Ct values when normalizing to secreted cells, UCMSCs (**Figure 2B**). Comparing the effects among different cytokines in each EV sub-populations, IFNα and TNFα cytokine treatments further limited the miR-181b-3p content in ABs and MVs (**Figure 2B**). Indeed, we detected a greater relative expressionof miR-181b-3p in TGFβ-ABs compared to IFNα-ABs (*p* = 0.0359) and TNFα-ABs (*p* = 0.0101), as well as in TGFβ-MVs compared to TNFα-MVs (*p* = 0.028). miR-181b-3p also expressed stronger in MVs from IFNα-primed UCMSCs compared to TNFα-primed ones (*p* = 0.038) (**Figure 2B**). However, in the EX population, no significant difference in miR-181b-3p inhibiting effects was observed among three different cytokine priming conditions (**Figure 2B**). Meanwhile, the amount of miR-320a-3p packed in ABs, MVs, and EXs was mostly stable in all non-priming and cytokine-priming cultures. Only a small change of miR-320a-3p content in the EX population was detected under the effect of TGFβ and TNFα, where this miRNA was relatively expressed higher in CT-EXs and TNFα-EXs compared to TGFβ-EXs (*p* = 0.0299 and *p* = 0.0031, respectively) (**Figure 2A**). Taken together, cytokine-primed UCMSCs selectively reduced the sorting of miR-181b-3p into EVs, whereas there were no effects on the miR-320a-3p.

### Chondrocytes were successfully isolated from human articular cartilage

**Figure 3:**
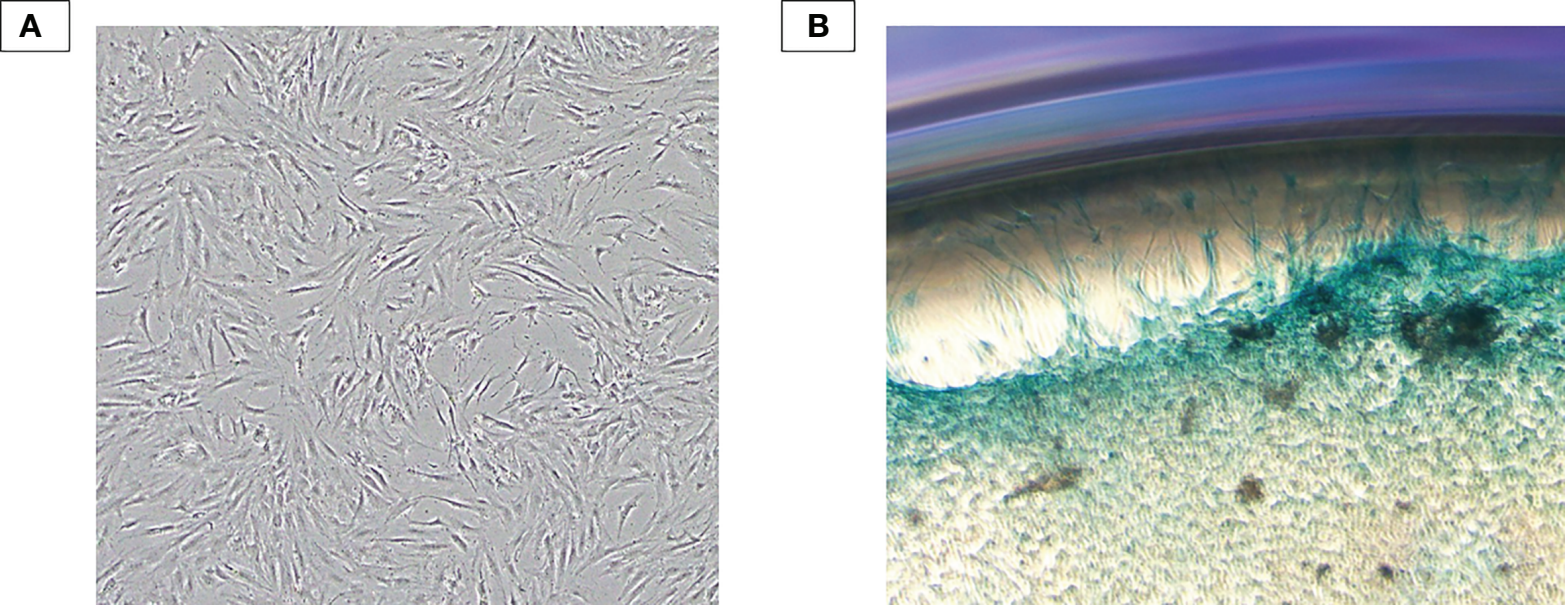
Chondrocyte characterization. **(A)** Human primary chondrocytes isolated from the human knee articular cartilage tissue at P1. Isolated cells exhibited typical morphology of primary chondrocytes: flattened and polygonal shape. **(B)** Alcian Blue staining result. Isolated cells were subjected to form colony and positive with Alcian Blue staining dye – an indicator of proteoglycan secretion.

Human chondrocytes were isolated from the knee articular cartilage tissue digested with collagenase and were cultured in the DMEM/F12. As shown in **Figure 3A**, cells at P1 exhibited flattened and polygonal shape which is a typical morphology of chondrocytes. Additionally, isolated cells were positive with Alcian Blue staining dye, which is specific for chondrocyte cells and appears blue due to proteoglycans secretion (**Figure 3B**).

### Cytokines affecting UCMSC-derived EV capacity in promoting chondrocyte proliferation

**Figure 4:**
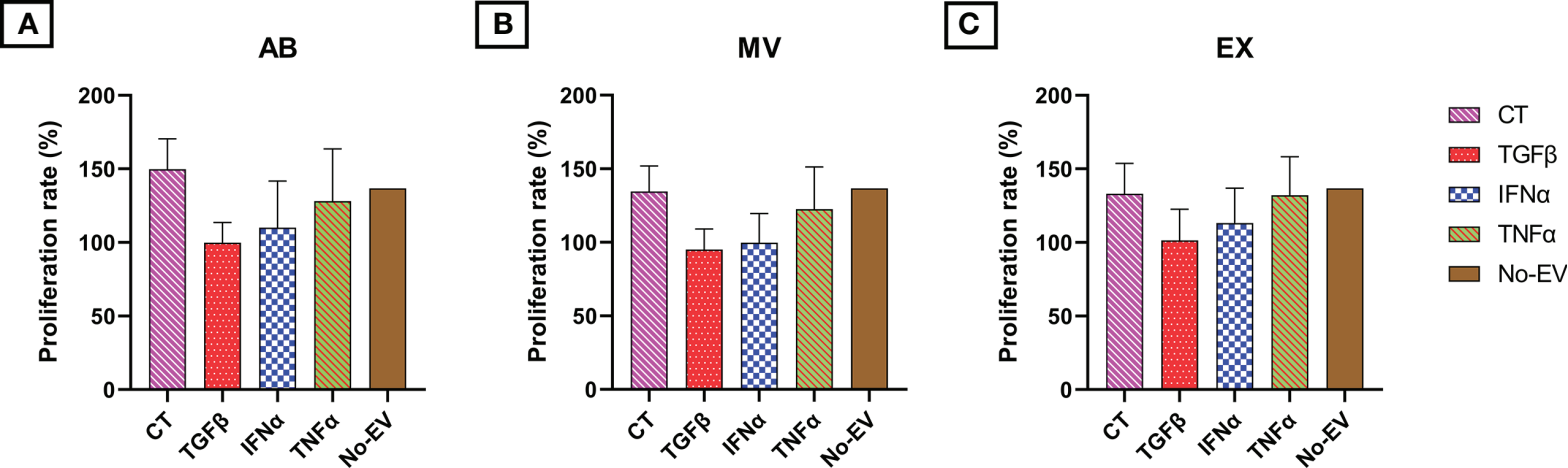
The influence of EV treatment on chondrocyte proliferation at 48h. Chondrocyte proliferation under the treatment of **(A)** AB populations; **(B)** MV populations; **(C)** EX populations. No statistically significant effect was observed among all EV treatment groups. CT-AB/MV/EX: chondrocytes treated with AB/MV/EX secreted from non-priming UCMSCs; TGFβ-AB/MV/EX: chondrocytes treated with AB/MV/EX secreted from TGFβ-primed UCMSCs; IFNα-AB/MV/EX: chondrocytes treated with AB/MV/EX secreted from IFNα-primed UCMSCs; TNFα-AB/MV/EX: chondrocytes treated with AB/MV/EX secreted from TNFα-primed UCMSCs; No-EV: chondrocytes cultured in DMEM/F12 5% EV-depleted FBS and no EV addition. The proliferation rate was acquired by normalizing absorbance measured at time point of 48 hours after incubation to absorbance measured at seeding time (0h), and the data were obtained from three independent biological replicates (n = 3). Statistical significance was determined by Two-Way ANOVA. Error bars indicate ± SD.

We performed an MTT assay on human chondrocytes to examine the effect of EVs derived from cytokine-priming UCMSCs on chondrocyte proliferation. In general, EVs generated from UCMSCs, either priming with cytokines or not, did not have any statistically significant effect on chondrocyte proliferation compared to EV-depleted media (No-EV) and among treatment groups (**Figure 4**).

### EVs derived from TGFβ-primed UCMSCs promoted chondrocyte migration

**Figure 5:**
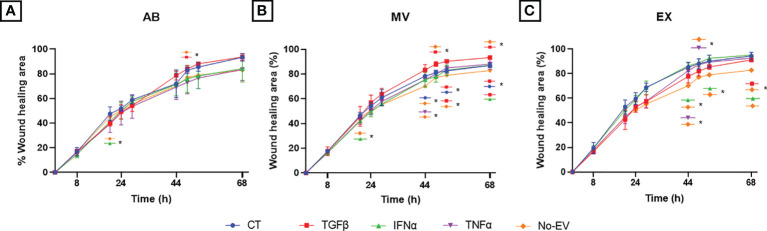
Priming UCMSCs with cytokines differentially affected the capacity of derived EVs to stimulate chondrocyte migration. Chondrocyte migration was analyzed using a wound-scratch assay under the treatment of **(A)** UCMSC-ABs **(B)** UCMSC-MVs; **(C)** UCMSC-EXs. TGFβ-EVs induced chondrocyte migration significantly at latter experimental time points. CT-AB/MV/EX: chondrocytes treated with AB/MV/EX secreted from non-priming UCMSCs; TGFβ-AB/MV/EX: chondrocytes treated with AB/MV/EX secreted from TGFβ-primed UCMSCs; IFNα-AB/MV/EX: chondrocytes treated with AB/MV/EX secreted from IFNα-primed UCMSCs; TNFα-AB/MV/EX: chondrocytes treated with AB/MV/EX secreted from TNFα-primed UCMSCs; No-EV: chondrocytes cultured in DMEM/F12 5% EV-depleted FBS and no EV addition. The images were captured at different time points in which chondrocytes migrated by time to close the wound and analyzed using ImageJ. Data are presented as the mean percent area of wound coverage in μm^2^ ± SD (n = 3). Statistical significance was determined by Two-Way ANOVA and indicated by: * where *p* < 0.05. Error bars indicate ± SD.

We performed the wound scratch assay to access the capacity of EVs from cytokine-primed UCMSCs in regulating chondrocyte migration. In general, EVs from either non-priming or cytokine-primed UCMSCs significantly promoted chondrocyte migration starting from the 44-hour time point (later experimental time points) (CT-ABs, *p* = 0.0445 at 68 hours; and CT-MVs, *p* = 0.0105 at 44 hours; when compared to EV-depleted media) (**Figure 5** and **Supplementary Figure 2**).

Among analyzed cytokines, EVs derived from TGFβ-primed UCMSCs significantly stimulated cell migration stronger than EV-depleted media at multiple time points. For details, TGFβ-ABs had higher migration induction at 52 hours (*p* = 0.0169) (**Figure 5A**). TGFβ-MVs enhanced migration at 48 hours (*p* = 0.0329), 52 hours (*p* = 0.0108), and 68 hours (*p* = 0.0129) (**Figure 5B**). Additionally, TGFβ-MVs exhibited a greater induction on cell migration than CT-MVs at 52 hours (*p* = 0.0215) and at 68 hours (*p* = 0.0424) and better than IFNα-MVs at 68 hours (*p* = 0.0426) (**Figure 5B**). At 68 hours, TGFβ-EXs significantly promoted migration stronger than No-EV (*p* = 0.0351) (**Figure 5C**).

Regarding IFNα cytokine priming, only IFNα-EXs induced cell migration faster than No-EV, but neither IFNα-ABs nor IFNα-MVs, at 44 hours (*p* = 0.0319), 52 hours (*p* = 0.0376), and 68 hours (*p* = 0.0338) (**Figures 5A, B**). Especially, IFNα-ABs and IFNα-MVs showed suppression of chondrocyte migration at the 20-hour time point (compared to No-EV; *p* = 0.0259 and *p* = 0.0190, respectively) (**Figures 5A, B**).

For EVs secreted from TNFα-primed UCMSCs, TNFα-MVs stimulated migration more effectively than No-EV at 44 hours (*p* = 0.0456), whereas TNFα-EXs promoted efficiently at 44 hours (*p* = 0.0492) and 48 hours (*p* = 0.0128) (**Figures 5B, C**).

### EVs derived from cytokine-primed UCMSCs alter the expression of chondrocyte markers by chondrocytes

To investigate the molecular alterations of chondrocytes in different EV-treated culture conditions, we isolated total RNA from cells after one-week culture under EV treatment and subjected them to qRT-PCR. The relative expression level of chondrocyte mRNAs, including normal chondrocyte markers of Collagen type II (COL2A1), Cartilage oligomeric matrix protein (COMP), Aggrecan (ACAN), and hypertrophic chondrocyte markers of Collagen type I (COL1A1), Runt-related transcription factor 2 (RUNX2), were calculated and represented as fold change.

**Figure 6:**
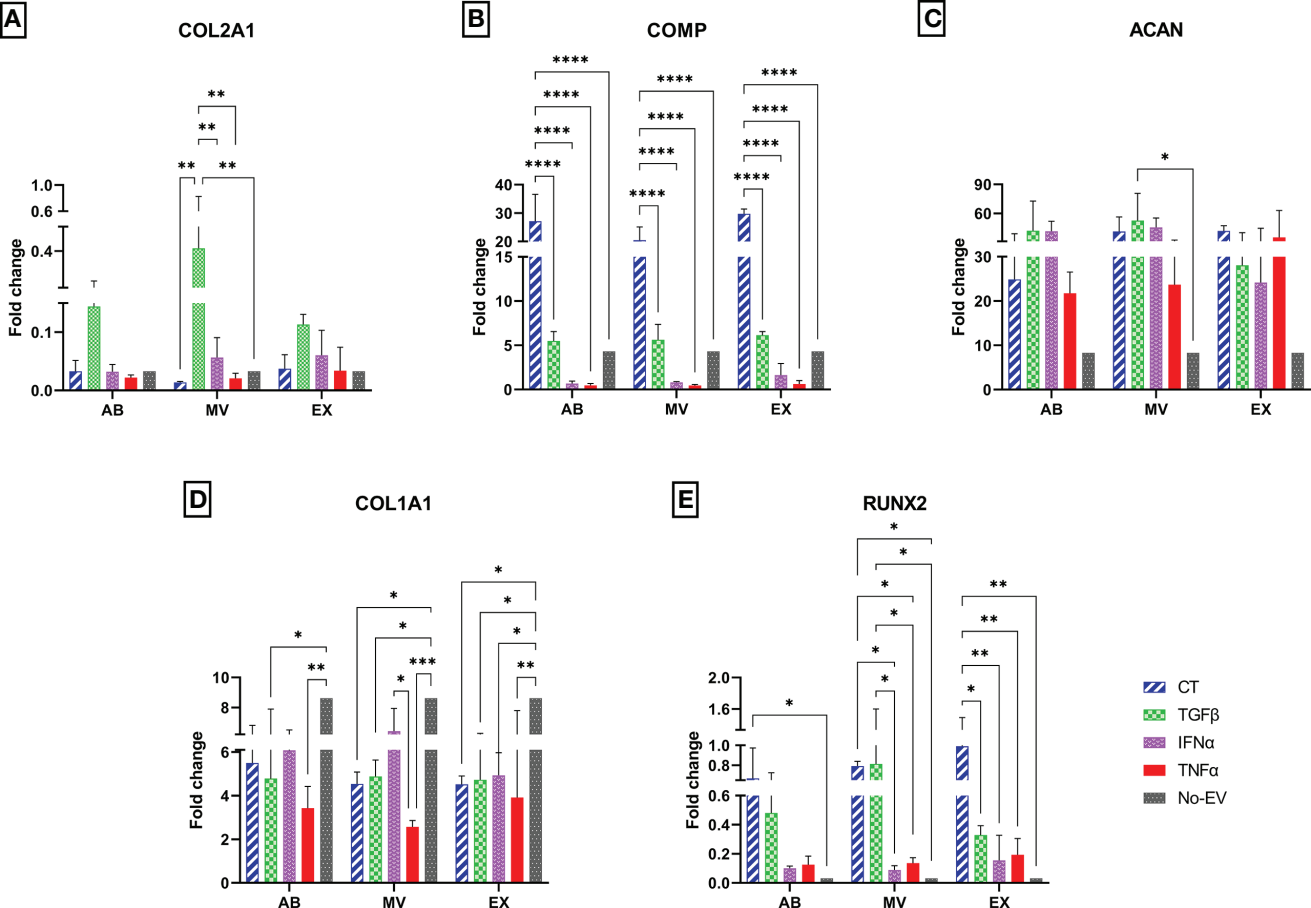
Different expression profiles of chondrocyte molecular markers. The mRNA relative expression levels in chondrocytes at passage 7 are represented by 2^-ΔΔCt^ values with 10 ng cDNA input and were normalized to the reference gene GAPDH and chondrocytes P3 cultured in DMEM 10% FBS. The relative expression level of **(A)** *COL2A1*; **(B)** *COMP*; **(C)** *ACAN*; **(D)** *COL1A1*; **(E)** *RUNX2*. Chondrocytes treated with EVs from TGFβ-primed UCMSCs expressed stronger *COL2A1* and lower *COL1A1*. AB: Apoptotic bodies; MV: Microvesicles; EX: Exosomes. CT-AB/MV/EX: chondrocytes treated with AB/MV/EX secreted from non-priming UCMSCs; TGFβ-AB/MV/EX: chondrocytes treated with AB/MV/EX secreted from TGFβ-primed UCMSCs; IFNα-AB/MV/EX: chondrocytes treated with AB/MV/EX secreted from IFNα-primed UCMSCs; TNFα-AB/MV/EX: chondrocytes treated with AB/MV/EX secreted from TNFα-primed UCMSCs; No-EV: chondrocytes cultured in DMEM/F12 5% EV-depleted FBS and no EV addition. Results were averaged of 3 biological replicates (n = 3). Statistical significance was determined by Two-Way ANOVA and indicated by: * where *p* < 0.05; ** where *p* < 0.01; *** where *p* < 0.001; **** where *p* < 0.0001. Error bars indicate ± SD.

In general, the treatment with either normal EVs or EVs associated with cytokine priming acted differentially on the expression of chondrocyte markers. We observed the highest expression of *COL2A1* in chondrocytes treated with TGFβ-MVs in all experimental groups (**Figure 6A**). Notably, non-priming EVs greatly enhanced the expression of *COMP* in chondrocytes, much stronger than any studied groups (**Figure 6B**). When analyzing the expression of *ACAN*, we observed that treatment with TGFβ-MVs significantly upregulated *ACAN* mRNA expression by chondrocytes compared to EV-depleted media (*p* = 0.0156) (**Figure 6C**).

Regarding hypertrophic markers, treating chondrocytes with EVs from both cytokine-primed and non-priming groups suppressed *COL1A1*. The downregulation of *COL1A1* was indicated by significantly lower expression levels in chondrocytes treated with CT-MVs and CT-EXs (*p* = 0.0195, and *p* = 0.0185, respectively); all three TGFβ-EV sub-populations (TGFβ-ABs, *p* = 0.0135; TGFβ-MVs, *p* = 0.0373; and TGFβ-EXs, *p* = 0.0282), IFNα-EXs (*p* = 0.0411), all three TNFα-EV sub-populations (TNFα-ABs, *p* = 0.0019, TNFα-MVs, *p* = 0.0003; and TNFα-EXs, *p* = 0.0054) (**Figure 6D**). Interestingly, the inhibition of *COL1A1* mRNA was further emphasized in the chondrocytes treated with TNFα-EVs, showed by a reduced expression of *COL1A1* by chondrocytes treated with TNFα-MVs compared to IFNα-MVs (*p* = 0.0310) (**Figure 6D**). In contrast with *COL1A1*, the expression of *RUNX2* was upregulated by chondrocyte treatment with EVs in general. Indeed, compared to cells cultured in EV-depleted media, *RUNX2* was expressed higher in chondrocytes treated with non-priming (CT) EVs (CT-ABs, *p* = 0.0498; CT-MVs, *p* = 0.0128; and CT-EXs, *p* = 0.0012), TGFβ-MVs (*p* = 0.01) (**Figure 6E**). However, priming cells with cytokines seemed to diminish this undesirable effect with lower expression of *RUNX2* in chondrocytes treated with MVs and EXs from IFNα- and TNFα-primed cells compared to chondrocytes treated with CT-EVs and TGFβ-EVs (**Figure 6E**).

Taken together, treatment of chondrocytes with cytokine-primed EVs partially rescued the chondrocytes from hypertrophic phenotype and established the primary, normal physical state of the cells.

## Discussion

In recent years, several techniques have been developed to optimize the therapeutic efficacy of EVs. Evidently, adding cytokines, such as IFNγ, TNFα, and IL1β, to the conventional MSC culture media affected the contents and biological activities of the derived EVs associated with OA (17, 23). Therefore, we investigated the influence of EVs derived from MSCs primed with anti-inflammatory (TGFβ and IFNα) and inflammatory (TNFα) cytokines on osteoarthritic chondrocytes. We found that cytokine priming did not affect the typical morphology and markers of UCMSCs. Additionally, the secreted EVs displayed distinguished sizes, morphologies, as well as surface markers (CD9 and CD63), which were in accordance with ISEV guidelines (24). These characteristics are also described in the previous studies of EVs from cytokine-primed UCMSCs (25, 26). This information allowed us to ensure the normal EV identity and further study the molecular profile and therapeutic effects of EVs under cytokine stimulations. Furthermore, stimulation of secreted cells with cytokines can also increase the amount of EVs produced by UCMSCs (14), which can potentially enrich therapeutic efficacy. Hence, the assessment of EV production from cytokine-stimulated UCMSCs should be considered in our future investigation.

As mentioned, the treatment of UCMSCs with various stimuli could affect the biological contents of EVs, including miRNAs, which have been reported for their potential roles in OA treatment (27, 28). Especially, it is emphasized that cytokine treatment can result in EVs with rich RNA profiles for inflammatory control (15), which can further reverse OA condition. This study reported the detection of miR-320a-3p and miR-181b-3p, which are involved in healthy cartilage maintenance and OA pathogenesis (18–20) in MSCs and three EV sub-populations released by UCMSCs. Our result indicates that miR-320a-3p was expressed higher in non-priming UCMSCs, while the expression level of miR-181b-3p was similar among groups. However, cytokines treatment diminished the packaging of miR-181b into EVs, shown by a significantly low relative expression of this EV miRNA associated with cytokine priming when normalized to the levels in UCMSCs. In literature, miR-181b promoted the NF-κB pathway, which leads to cartilage destruction and synovium membrane degradation (29–31). Blocking miR-181b activity reduced MMP13 expression but increased COL2 expression in articular chondrocytes (32). The attenuation of miR-181b activity can indirectly signal the FPR2- formyl peptide receptor and induce anti-inflammatory effects (33, 34). Thus, the reduction of miR-181b observed in EVs originating from cytokines-primed cells can be a positive marker for re-adjusting the appropriate EV components to produce more direct effects in cartilage regeneration. This exciting information requires further studies to validate whether two cytokines, IFNα and TNFα, could be the appropriate stimulus to enhance the therapeutic efficacy of UCMSC-EVs for OA treatment.

On the other hand, the level of miR-320a-3p remained stable across experimental EV treatments. Previous studies showed pieces of diverse evidence of miR-320a function in cartilage homeostasis (18, 19, 35). Peng et al. (19) also demonstrated the protective effects of miR-320a over cartilage degeneration by negatively regulating BMI-1 (19). However, miR-320a has also been shown as a potential OA marker as this miRNA promoted OA-induced matrix breakdown *via* the NF-κB pathway and interfered with osteoblast reformation (18, 35, 36). Thus, a future study is required to evaluate the roles of miR-320a-3p in OA pathogenesis and examine an alternative approach to adjusting this miRNA content in UCMSC-derived EVs.

Next, to examine our EVs’ bioactivity *in vitro*, we isolated human primary chondrocytes from articular cartilage tissues obtained from a patient suffering from a knee injury and performed proliferation, migration and mRNA markers analysis assays. Our isolated cells showed typical chondrocyte morphology and were positive with specific staining dye for proteoglycan. For functional analysis, in general, all EVs from either non-priming or cytokines-primed UCMSCs at the dose of 10 μg/mL did not promote chondrocyte proliferation significantly. This result may be due to an insufficient dose of EVs might be the issue, as higher doses (20, 40, 80 µg/mL) of BMMSC-EXs have been shown to increase the proliferation rate of chondrocytes (37). Additionally, the outcomes may be due to chondrocytes obtained from patients with knee injury reported herein instead of healthy chondrocyte cell lines. Hence, further experiments with chondrocytes induced with OA characteristics and higher EV dosage will be conducted to examine these possibilities. Notably, the miRNA distribution in EVs is an essential factor that might affect chondrocyte proliferation and migration; however, the regulation of miR-181b on these two biological processes remains unclear. A member of the miR-181 family, miR-181a, exerted adverse effects on chondrocyte proliferation by upregulating the expression of caspase-3, PARP, MMP-2, and MMP-9 to induce apoptosis and cartilage destruction (20). Thus, it is predicted that miR-181b can inhibit chondrocyte proliferation, and suppressing miR-181b expression can restore this ability. However, in this current study, the reduction in miR-181b might not contribute to proliferation results observed here, or other factors have surpassed its influence.

Contrary to cell proliferation, EVs from cytokines-primed UCMSCs expressed a higher capacity to promote chondrocyte migration compared to chondrocytes cultured in EV-depleted media, with the most significant effect belonging to TGFβ-EVs but only in later time points. In the previous study, TGFβ was shown to promote the PI3K-Akt signaling pathway, which was demonstrated to induce chondrocyte migration in a rat model (38, 39). Additionally, TGFβ stimulation can also regulate the integrin signaling pathway involving changes in integrin-ECM binding and the activation of FAK, which are critical factors in cell migration (40, 41). It is noted that all results obtained herein were in the comparison with chondrocytes cultured in EV-depleted media (DMEM/F12 supplemented with 5% EV-depleted FBS) but not as in most studies used PBS or chamber consisting of low serum media (upper) and PBS (lower) as the control group (42–44). Indeed, long-term cell storage with PBS increases cell death and thus cannot access cell functionality efficiently. Those factors may be reasons for the differences observed in this study compared to others.

In this study, we investigated the alteration in mRNA levels of chondrocyte markers under the treatment of EVs at high passage culture. The later culture passage exhibited an increase in *COL1* and a decrease in *COL2*. However, higher expression of healthy chondrocyte markers, including *COMP* and *ACAN*, was also detected, which supports cartilage regeneration and ECM synthesis at the later stage. Meanwhile, the expression of hypertrophic markers such as *RUNX2* diminished. Besides, we observed that EVs from cytokines-primed UCMSCs downregulated the expression of *COL1* and *RUNX2* and upregulated *COL2* and *ACAN* expression, but this effect was not consistent among EV populations. MiR320a was previously linked with low expression of hypertrophic marker RUNX2 (19). However, a stable level of miR-320a-3p in most of the isolated EVs from both non-priming and cytokine-primed UCMSCs hinders us from revealing the association between EV contents and chondrocyte markers. Notably, the increase in *COMP* expression was much more substantial in chondrocytes treated with EVs derived from non-priming UCMSCs. This means that EVs contribute to chondrocyte malfunctions or the recovery of damaged chondrocytes. However, further experiments, especially on miRNAs and target mRNAs, should be investigated to understand the mechanism of the effect of EV contents on chondrocyte mRNA expression.

In conclusion, cytokines influenced the miRNA composition of UCMSCs-derived EVs and their effects on chondrocyte physiology regarding cell proliferation and migration, as well as chondrocyte markers. However, it is noted that the results presented here are preliminary data that require more investigations on other miRNAs/proteins found in EVs in addition to the target genes and signaling pathways affecting the chondrocyte bioactivities. Additionally, for future perspectives, studies should be performed to examine the roles of different cytokines on UCMSC-derived EVs and their cargos in other aspects of OA, such as chondrocyte apoptosis and inflammation.

## Experimental procedures

### Ethical approval

Ethical approval for collecting and using human MSCs from the umbilical cord and human chondrocytes from articular cartilage was issued by the Vinmec International General Hospital Joint Stock Company’s ethics committee (Ethical approval number: 311/2018/QĐ-VMEC). The umbilical cord tissues were collected from three healthy donors aged 20 to 40, and human cartilage tissues were acquired from three donors with knee arthroplasty. Donors signed written informed consent before donating their samples.

### Umbilical cord-derived mesenchymal stem cell culture

UCMSCs were isolated from the umbilical cord following what was described in our previous study and stored for further experiments (45). UCMSCs at passage two (P2) were thawed and seeded at a density of 5,000 cells/cm^2^ in DMEM/F12 (Gibco, Massachusetts, USA) with 10% (v/v) fetal bovine serum (FBS). Cells were incubated in 37°C/5% CO_2_ condition and subcultured with the same density until passage 5 (P5). The cells at P5 were cultured with EV-depleted media for three days prior to cytokine treatments (DMEM/F12 supplemented with 10% EV depleted-FBS, in which FBS was centrifuged at 120,000 × *g* for 18 hours at 4°C to eliminate EVs). Cells were maintained in EV-depleted media before exposing to cytokines individually for 48 hours with the following concentrations: 10 ng/mL TGFβ, 20 ng/mL IFNα, or 20 ng/mL TNFα. The conditioned media were harvested when cells reached 95% confluency for EV isolation (cell culture media were not renewed throughout incubation). After conditioned media collection, UCMSCs were characterized with Human MSC Analysis Kit (BD Biosciences) following the manufacturer’s protocol, and flow cytometry data were analyzed with Navios Software 3.2.

### Extracellular vesicle isolation

The conditioned media was centrifuged at 300 × *g* for 10 minutes at 4°C to remove cell debris. Sequential centrifugation steps were performed to separate three EV populations as follows: 2,000 × *g* for 20 minutes at 4°C to collect apoptotic bodies (ABs), 16,500 × *g* for 30 minutes at 4°C to pellet microvesicles (MVs), and 100,000 × *g* for 90 minutes at 4°C for isolation of exosomes (EXs) (Optima XPN-100 Ultracentrifuge, Beckman Coulter, California, USA). EV pellets were resuspended in DMEM/F12 or PBS and stored at − 80°C for further usage.

### Extracellular vesicle marker analysis by western blot

Protein extraction and western blot were performed as described previously (45). Total EV protein concentrations were determined by Pierce™ BCA Protein Assay Kit (Thermo Scientific, Massachusetts, USA) and as equivalent to an optical density (OD) measured at 562 nm (SpectraMax M3, Molecular Devices, California, USA). Then, 15 µg of EV proteins were electrophoretically separated by 4 – 12% NuPAGE gels (Invitrogen, Massachusetts, USA) and probed with primary antibodies (Abcam, Cambridge, UK) against GAPDH, CD9, and CD63 overnight at 4°C, followed by the incubation with goat anti-Rabbit IgG secondary antibody (Invitrogen, Massachusetts, USA). Antibody binding was stained with ECL substrate and visualized on ImageQuant LAS 500 (GE Healthcare Life Sciences, Illinois, USA).

### Extracellular vesicle morphology analysis by transmission electron microscopy

EV samples were fixed and stained following the protocol described in our previous study (45). Imaging was performed using a JEOL 1100 Transmission Electron Microscope (JEOL Ltd., Tokyo, Japan) at 80 kV at the National Institute of Hygiene and Epidemiology (NIHE).

### Chondrocyte isolation and characterization

Human cartilage tissues were collected by the surgical doctors, stored in saline water at 4°C, and transferred to the laboratory. Before processing, the tissue was washed once with ethanol 70%, twice with PBS, and once with DMEM/F12; each solution was supplemented with 1% Pen/Strep (Thermo Fisher Scientific, USA) to ensure sterile and eliminate contaminants. The tissue was minced and digested in Hanks’ Balanced Salt Solution (HBSS) (Thermo Fisher Scientific, USA) 0.2% collagenase type I 10000 U/mL solution (Gibco, Massachusetts, USA) (10 mL for every 1 gram of tissue) for 20 hours at 37°C. Cell culture media (DMEM/F12 supplemented with 10% FBS (v/v)) was added in a volume ratio of 1: 1 with HBSS. The harvested pellets were resuspended in DMEM/F12 supplemented with 1% Pen/Strep and 10% FBS (v/v), then seeded into a T25 cell culture flask and incubated at 37°C and 5% CO_2_. The media were replaced by every three days during the cultures. After reaching 80% confluency, the cells were either stored or subcultured at the density of 10,000 cells/cm^2^ to the next passage.

The images of chondrocytes were captured under Eclipse Ti-S Inverted Microscope (Nikon Instruments, Japan), and cells at P0 were processed to form the colony and stained with Alcian Blue to confirm cell type.

### Total RNA extraction

Total RNA was extracted using Trizol™ reagent (Thermo Scientific, Massachusetts, USA) with a ratio of 9: 1 Trizol versus cell/particle suspension. The lysis mixture was added with MgCl_2_ and chloroform and incubated at RT. The aqueous phase was collected and incubated in with isopropanol overnight at -20°C. Total RNA was then pelleted with centrifugation and then washed twice with RNase-free 75% ethanol before air-drying and resuspending in RNase-free water (volume based on pellet size).

### Quantitative reverse transcription-PCR

Total RNA with sufficient quality was subjected to qRT-PCR to confirm the presence of EV miRNAs and chondrocyte mRNAs.

For EV miRNA analysis, extracted RNAs were used as templates to prepare cDNA using the miScript II RT kit (Qiagen, Hilden, Germany), following the manufacturer’s instructions. Then, cDNA-containing mixtures (10 μL) were subjected to qPCR using the miScript SYBR Green PCR kit (Qiagen, Hilden, Germany) and two specific primers, miScript Primer Assay 10X (Qiagen, Hilden, Germany) designed to target miR-320a-3p and miR-181b-3p. The incubation was performed on Applied Biosystems 7500 Block (Applied Biosystems, Massachusetts, USA). The relative expression of miRNAs in UCMSCs was normalized to reference gene RNU6B (Qiagen, Hilden, Germany) and miRNAs in EVs was normalized to their secreted cells (UCMSCs) and represented by the ΔCt values, with a higher ΔCt value representing a less selective sorting of this miRNA into EVs and vice versa.

For chondrocyte mRNAs analysis, cells were cultured for one week under treatment as described in **Table 1**, and chondrocyte RNAs were isolated as above. cDNA was prepared using SuperScript™ IV Reverse Transcriptase (Thermo Scientific, Massachusetts, USA), and step by step was performed according to the manufacturer’s protocol. cDNA products were then subjected to qPCR reaction, using specific-designed primers that targeted chondrocyte RNAs, including normal chondrocyte markers of Collagen type II (COL2A1), Cartilage oligomeric matrix protein (COMP), and Aggrecan (ACAN) and hypertrophic chondrocyte markers of Collagen type I (COL1A1) and Runt-related transcription factor 2 (RUNX2), and *GAPDH* as an internal control (primer sequences were listed in **Supplementary Table 1**). 2^-ΔΔCt^ method was applied to calculate the relative fold gene expression of samples.

**Table 1:** Experimental settings for chondrocyte functional analysis *in vitro*.

Experimental Groups	Description
CT-AB/ MV/ EX	Chondrocytes cultured in DMEM/F12 5 % EV-depleted FBS supplemented with 10 μg/mL AB/ MV/ EX from non-priming UCMSCs
TGFβ-AB/ MV/ EX	Chondrocytes cultured in DMEM/F12 5 % EV-depleted FBS supplemented with 10 μg/mL AB/ MV/ EX from TGFβ-primed UCMSCs
IFNα-AB/ MV/ EX	Chondrocytes cultured in DMEM/F12 5 % EV-depleted FBS supplemented with 10 μg/mL AB/ MV/ EX from IFNα-primed UCMSCs
TNFα-AB/ MV/ EX	Chondrocytes cultured in DMEM/F12 5 % EV-depleted FBS supplemented with 10 μg/mL AB/ MV/ EX from TNFα-primed UCMSCs
No-EV	Chondrocytes cultured in DMEM/F12 5 % EV-depleted FBS

The amount of EVs used in experiments was determined based on our previous study (45).

### Proliferation assay

Human articular chondrocytes were seeded (2,500 cells in each well of 96-well-plate) and incubated in media as listed in **Table 1**. No-EV was used as the control. Cells were incubated at 37°C and 5% CO_2_ for 48 hours to proliferate. The cell proliferation rate was assessed by performing a 3-(4,5-dimethylthiazol-2-yl)-2,5-diphenyl tetrazolium bromide (MTT) assay (Abcam, Cambridge, UK) following the manufacturer’s protocols. The proliferation rate was equivalent to the relative absorbance measured at 562 nm (SpectraMax M3, Molecular Devices, California, USA) at time points of 0 hours (as used for normalization) and 48 hours. The proliferation rate was calculated based on the OD values obtained from two time points.

### Migration assay

Human articular chondrocytes were cultured in a 24-well plate with a density of 1.05 × 10^5^ cells/well at 37°C and 5% CO_2_ for attachment. After reaching 100% confluency, cells were then incubated with Mitomycin C (10 μg/mL) for 2 hours to inhibit cell proliferation. A physical scratch was created on the cell attachment layer, and detached cells were removed by washing with media. Treatments were established similarly to proliferation assay (**Table 1**). Cell migration to close the wound area was captured by an inverted microscope at multiple time points. The wound area was measured using ImageJ software (version 1.48) and calculated for the closure percentage over time, which represents the rate of cell migration.

### Statistical analysis

The statistical analysis was performed on GraphPad Prism 9 (GraphPad Software, California, USA) using One-Way and Two-Way ANOVA, and Tukey HSD tests. The statistical significance was defined as a *p*-value < 0.05. All data were shown as means ± SD of three biological replicates.

## Data availability statement

The original contributions presented in the study are included in the article/**Supplementary Material**. Further inquiries can be directed to the corresponding authors.

## Author contributions

The conception and design of the study: UT, LN, ThN, HD, and XHN. Analysis and interpretation of data: UT, LN, ThN, HD, XHN, HD, TT, TN, CD, and H-XD. Manuscript drafting: ThN, HD and CD. Manuscript revising: UT, XHN. Final approval: UT. All authors contributed to the article and approved the submitted version.

## Funding

This project was funded by Vietnam Ministry of Health with decision number 2575/QĐ-BYT (20/06/2019) and Vinmec Joint Stock Company (ISC.18.07). This source has no involvement in the study design, collection, analysis and interpretation of data in the writing of the manuscript and in the decision to submit the manuscript of publication.

## Acknowledgments

We thank the doctors and nurses from Hanoi Medical Hospital for cartilage collection.

## Conflict of interest

The authors declare that the research was conducted in the absence of any commercial or financial relationships that could be construed as a potential conflict of interest.

## Publisher’s note

All claims expressed in this article are solely those of the authors and do not necessarily represent those of their affiliated organizations, or those of the publisher, the editors and the reviewers. Any product that may be evaluated in this article, or claim that may be made by its manufacturer, is not guaranteed or endorsed by the publisher.
