# Supplementary material for: Cytokine-primed umbilical cord mesenchymal stem cells enhanced therapeutic effects of extracellular vesicles on osteoarthritic chondrocytes
Source: Front Immunol. 2022 Oct 27;13:1041592. doi: 10.3389/fimmu.2022.1041592 (PMC9647019; doi:10.3389/fimmu.2022.1041592)
Supplement: Supplementary file 1 [file DataSheet_1.docx]

**Supplementary Figure 1: miR-320a-3p and miR-181b-3p** **expression in parental cells.**

The miRNA expression levels in parental UCMSCs are represented by delta Ct values with 7.5 ng cDNA input and were normalized with reference gene RNU6B. The relative expression level of **(A)** miR-320a-3p and **(B)** miR-181b-3p in UCMSCs cultured in different conditions. Non-priming UCMSCs had the lowest expression levels of miR-320a-3p while the expression of miR-181b-3p was similar among tested groups. CT: non-priming UCMSCs; TGFβ: TGFβ-primed UCMSCs; IFNα: IFNα-primed UCMSCs; TNFα: TNFα-primed UCMSCs. Results were averaged of 3 biological replicates (n = 3). Statistical significance was determined by One-Way ANOVA and indicated by ** where *p* < 0.01; *** where *p* < 0.001; **** where *p* < 0.0001. Error bars indicate ± SD.

**Supplementary Figure 2**: Representatives of human chondrocyte migration under microscopy at different time intervals 0h, 8h, 24h, 44h, and 68h.

The images were captured at different time points in which chondrocytes migrated by time to close the wound and analyzed using ImageJ. CT-AB/ MV/ EX: chondrocytes treated with AB/ MV/ EX secreted from non-priming UCMSCs; TGFβ-AB/ MV/ EX: chondrocytes treated with AB/ MV/ EX secreted from TGFβ-primed UCMSCs; IFNα-AB/ MV/ EX: chondrocytes treated with AB/ MV/ EX secreted from IFNα-primed UCMSCs; TNFα-AB/ MV/ EX: chondrocytes treated with AB/ MV/ EX secreted from TNFα-primed UCMSCs; No-EV: chondrocytes cultured in DMEM/F12 5 % EV-depleted FBS and no EV addition.

| **Supplementary Table 1.** Primer sequences targeted ECM mRNAs | | |
| --- | --- | --- |
| **Gene Name** |  | **Primer Sequences (5' - 3')** |
| ***COL2A1*** | Forward | 5′-GGCCTCCCCGGAACTC-3′ |
|  | Reverse | 5′-CCTCTTCCGCCGTCTTTC-3′ |
| ***COMP*** | Forward | 5′-GGATGCCTGTGACAACTGTC-3′ |
|  | Reverse | 5′-AAGGCCCTGAAGTCGGTGAG-3′ |
| ***ACAN*** | Forward | 5′-CCAGGAGGTATGTGAGGA-3′ |
|  | Reverse | 5′-CGATCCACTGGTAGTCTTG-3′ |
| ***COL1A1*** | Forward | 5′-CCTCAAGGGCTCCAACGAG-3′ |
|  | Reverse | 5′-TCAATCACTGTCTTGCCCCA-3′ |
| ***RUNX2*** | Forward | 5′-TGCCTCATCCCTGCCTCTGTGT-3′ |
|  | Reverse | 5′-TGTGGGGCTGCTCAGGAGGG-3′ |
| ***GAPDH*** | Forward | 5'-GGTGTGAACCATGAGAAGTATGA-3' |
|  | Reverse | 5'-GAGTCCTTCCACGATACCAAAG-3' |
| COL2A1 (Collagen type II), COMP (Cartilage oligomeric matrix protein), ACAN (Aggrecan), COL1A1 (Collagen type I), RUNX2 (Runt-related transcription factor 2), and GAPDH (Glyceraldehyde 3-phosphate dehydrogenase) as an internal control. | | |
|  |  |  |
