# Supplementary figures and images for: Cytokine-primed umbilical cord mesenchymal stem cells enhanced therapeutic effects of extracellular vesicles on osteoarthritic chondrocytes
Source: Front Immunol. 2022 Oct 27;13:1041592. doi: 10.3389/fimmu.2022.1041592 (PMC9647019; doi:10.3389/fimmu.2022.1041592)

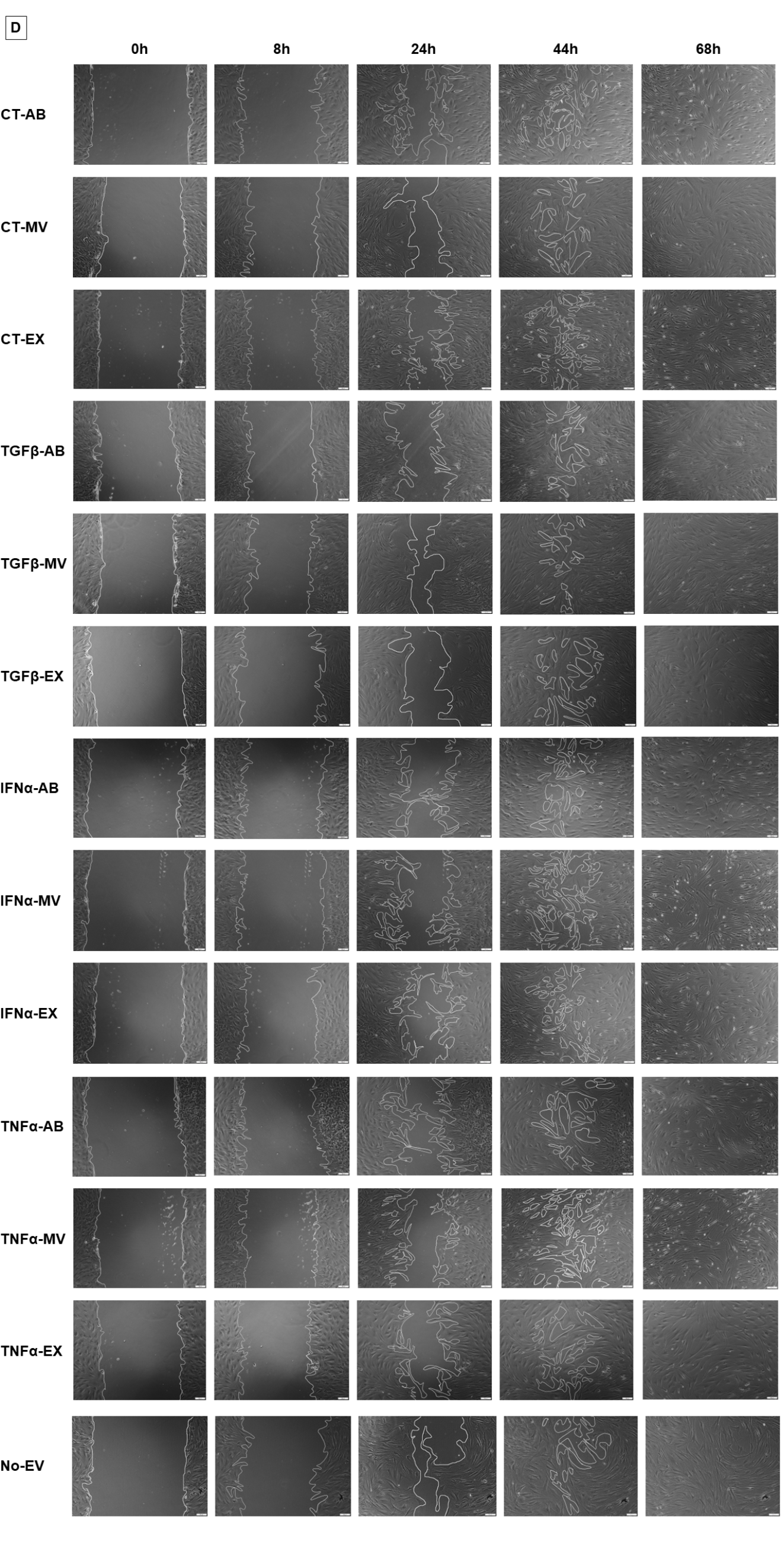

Supplement: Supplementary file 3 [file Image_2.tif]
